# Evolution with Reinforcement Learning in Negotiation

**DOI:** 10.1371/journal.pone.0102840

**Published:** 2014-07-21

**Authors:** Yi Zou, Wenjie Zhan, Yuan Shao

**Affiliations:** School of Management, Huazhong University of Science and Technology, Wuhan, P.R. China; University of Maribor, Slovenia

## Abstract

Adaptive behavior depends less on the details of the negotiation process and makes more robust predictions in the long term as compared to in the short term. However, the extant literature on population dynamics for behavior adjustment has only examined the current situation. To offset this limitation, we propose a synergy of evolutionary algorithm and reinforcement learning to investigate long-term collective performance and strategy evolution. The model adopts reinforcement learning with a tradeoff between historical and current information to make decisions when the strategies of agents evolve through repeated interactions. The results demonstrate that the strategies in populations converge to stable states, and the agents gradually form steady negotiation habits. Agents that adopt reinforcement learning perform better in payoff, fairness, and stableness than their counterparts using classic evolutionary algorithm.

## Introduction

Uncertainty in negotiation can be caused by a number of factors, such as fuzzy opponent type, unknown strategies, and deadlines [1]. Hence, one of the central topics in negotiation is how to design agents with higher adaptability for changing circumstances [2], [3]. In the environment of incomplete information, the primary concern is how to acquire more information and use it appropriately to reach consensus through concession in negotiation [4]–[6]. Through feedback, the agents learn how to make future decisions. Because the learning effect in the short term depends strongly on the details of the negotiation process and the characteristics of the opponent, it is difficult to evaluate a learning model through sparse interactions. In addition, because heterogeneous agents behave differently, it is reasonable to assess the performance from a macroscopic view [7], [8].

To address the uncertainty, learning in the long term emphasizes strategy selection and adjustment through repeated negotiations using qualitative or quantitative methods. For instance, Eduard Gim'enez-Funes et al. have applied a qualitative approach, case-based reasoning, to find the appropriate strategy by comparing the similarity of current situations to history [9]. Matos et al. have applied a widely used quantitative model, an evolutionary algorithm, to investigate long-term behavior [10]. This approach is derived from biology, with simple rules to evaluate payoff [11]. A number of researchers have investigated strategy evolution in the long term in recent years [12]–[17]. In the genetic algorithm, agents with higher fitness are passively selected and put into the mating pool to replicate the next generation, without considering the learning behavior of the agents. This approach myopically assesses the performance of agents with payoff only in the current period, while humans learn by weighing both the historical information and the current performance. If the agent represents a person in reality, it is reasonable to incorporate individual learning because humans adjust strategy through experience with initiative.

Fudenberg and Levine have investigated long-term strategy dynamics, including replicator dynamics and reinforcement learning [18]. Because reinforcement learning is a type of individual learning while the evolutionary approach concerns population dynamics, prior literature has generally examined them separately. However, Börgers, T. and R. Sarin [19] find that a type of continuous time reinforcement learning can converge to an equilibrium of replicator dynamics, which indicates some interaction between population dynamics and individual reinforcement learning. Reinforcement learning, with its basis in psychology [20], [21], evaluates the reward by weighting historical and current payoffs and has been applied to human strategy adjustment as well as to artificial intelligence [22]–[24]. Recently, researchers have begun to integrate different learning approaches to determine an agent's optimal strategy in the case of incomplete information. Reinforcement learning is a good fit when information on the opponent and environment is limited. To this end, agents in our model adopt reinforcement learning to calculate the reward of each strategy and then use replicator dynamics to adjust the probability of strategies. We integrate replicator dynamics and reinforcement learning to explore the efficiency, fairness, and strategy convergence in negotiation. In addition to the efficiency and strategy evolution, fairness has also been a concern of many researchers [25], [26]. The simulation results indicate that our approach achieves higher reward, shorter negotiation time, and a lower degree of greediness of strategies than the classic evolution model. It is also shown that the weight tradeoff between current and historical experience impacts the negotiation performance and learning effect to a large extent.

## Methods

**Figure 1:**
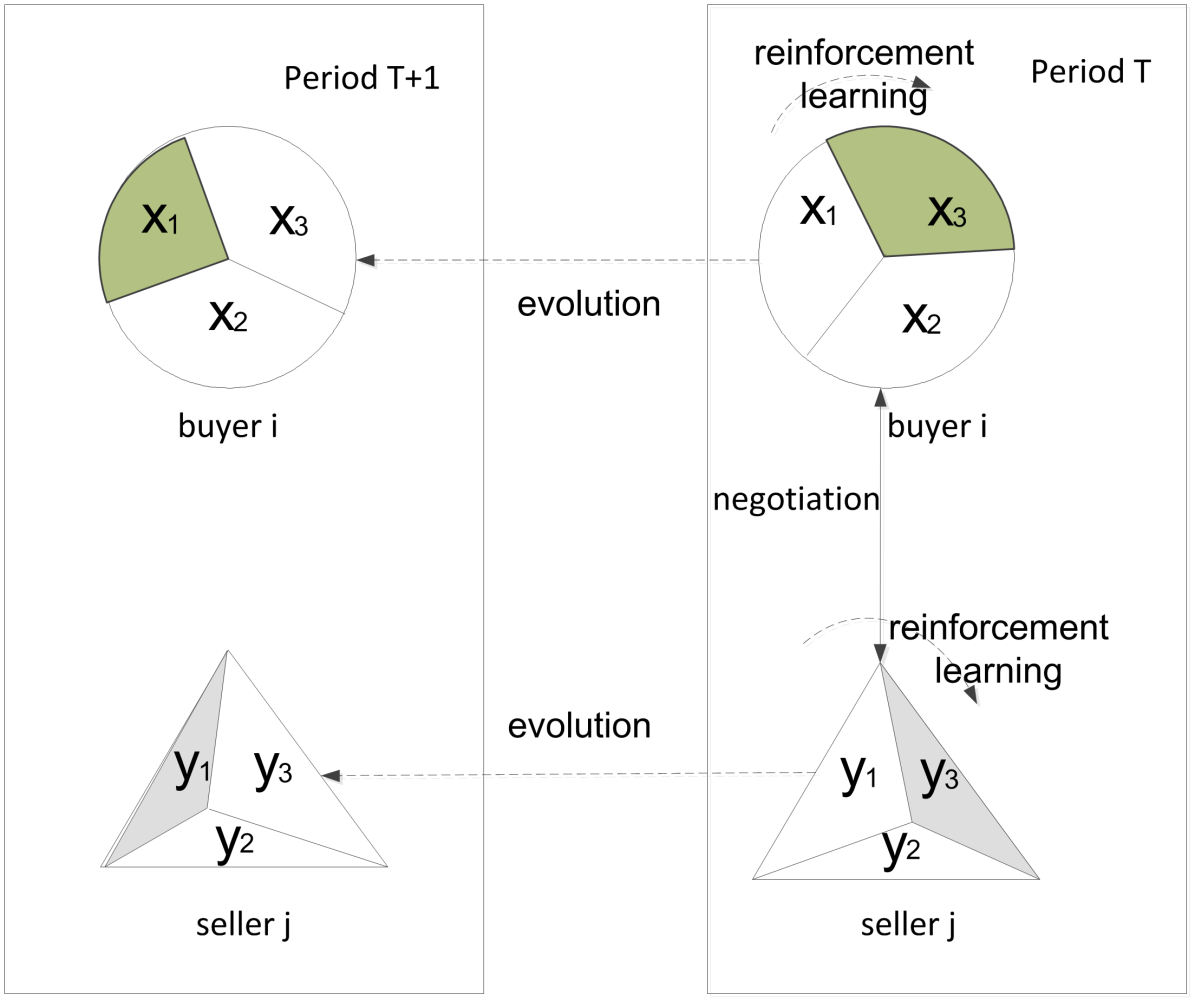
The negotiation model. The circles denote buyers; triangles denote sellers, and ellipses denote environmental factors. The values 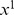,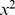,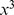 are probabilities of the frugal, cool-headed and anxious strategies of buyers, respectively, and 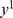,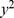,
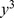
are the corresponding strategies for the sellers. The shaded area represents the selected strategy in the current period. At the end of each negotiation period, the buyers (sellers) calculate the reward through reinforcement learning and update 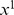,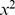,
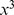
(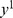,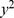,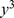) accordingly. The process continues until the strategies of all agents in the market converge to a stable state.

This model is based on the alternate offering mechanism of Rubinstein, in which agents adopt the time-dependent concession function [10]. The agents use reinforcement learning to accumulate experience and update the probability of each strategy using replicator dynamics. The negotiation process is illustrated in Figure 1.

### Negotiation rules

The reservation prices 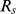 and 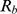 of the sellers and buyers are uniformly distributed between 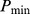 and 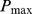. In each period, seller-buyer pairs are randomly selected and begin to negotiate if 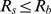. The offering intervals for a buyer and a seller are (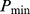,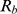) and (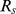,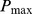), respectively. They alternate in making offers, and the discount rates of sellers and buyers are 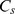 and 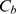, respectively (the first offer is proposed by the buyer by default). If an agent rejects the offer of the opponent, he then proposes his own offer (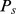 for the seller and 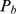 for the buyer), and the accepted price is *P*. The payoff of the seller is 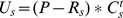, and the payoff of the buyer is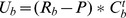, where t is the negotiation time.

### Concession functions

The time-dependent strategies for buyers and sellers are 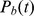 and 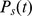, respectively, and the concession rate increases with time. 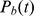 and 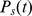
are represented in equation (1) as follows:
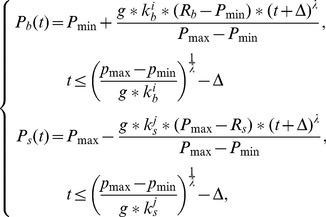
(1)where *g* denotes the time pressure of negotiators, and 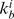,
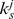
(0<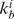, 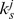
<1) denote the greediness of buyers and sellers, with smaller 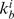 and 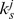 values indicating higher levels of greediness. Three strategies with different degrees of greediness exist for each population: 0<
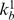
<
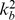
<
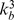
<1, 0<
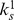
<
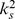
<
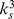
<1. Here, 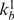 represents the frugal strategy; 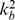 represents the cool-headed strategy; and 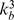 represents the anxious strategy for the buyers as well as for the sellers. 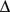 refers to the initial offer parameter, which creates an offer closer to the reservation price, with a larger 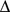; 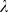 refers to the concession type, which is defined as a convex function for buyers and a concave function for sellers when 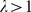. The buyer and the seller propose offers alternately until an offer is accepted, which occurs when the agent receives a higher payoff than refusing it by proposing his own offer, which is expected to be accepted by the opponent in the next round. The buyer accepts an offer 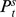 when 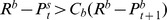, and a seller accepts 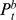 when 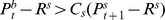.

### Learning rules

The agents update their rewards according to feedback based on historical information and eventually develop a negotiation habit [27]. Due to the constantly changing environment, the reference value of information decreases with time, and thus, the agents assign different weights to historical and current payoffs. The reward function is defined as follows:
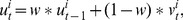
(2)where 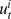 is the average reward of strategy
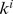
in period t, *w* is the weight of the historical payoff, and 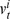 is the average current payoff strategy 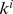. At first, each agent has a subjective probability for every strategy and thereby chooses one strategy to negotiate. In period t, the agent chooses strategy 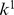 with probability 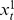, 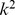 with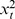, 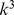 with 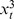, and 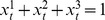. The agent updates the probabilities according to the rewards of each strategy, where a higher reward leads to an increased probability in the next period and vice versa. Until the total negotiation frequency reaches N, the agents adjust their strategy, which is defined as a learning period. The adjustment refers to replicator dynamics, but it is slightly modified to fit the reality:
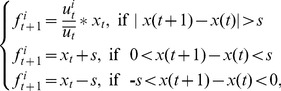
(3)




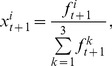
(4)where 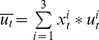 is the average payoff, and 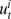 is the payoff of strategy 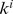 in period t. In Eq.(3), 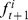 is the temporary probability of strategy i, and 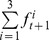
does not necessarily equal 1. In Eq.(4), 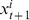 is the normalized result, which ensures 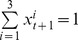. In period t+1, the probability of strategy 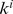 is proportional to 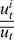, which means that the probability increases in the next period when 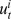 is more than 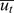 and decreases in the opposite case. We define the adjustment precision as *s* with the default value of 0.01. When the change in probability is smaller than *s* (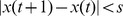), the adjustment size is *s*. When 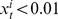, we set 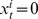, which means that this strategy has vanished. When 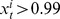, we set 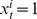, which means that the agent has converged to this strategy. In addition, 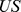 denotes the payoff of the seller without learning, and 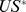 denotes the payoff with learning. Similarly, 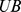 and 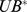 denote the corresponding meanings for the buyer. Fairness without learning is evaluated by 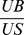, and fairness with learning is evaluated by 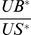.

### Experiments

The first experiment investigates the general performance of the agents when *g* = 6, 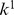 = 0.3,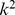 = 0.5,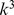 = 0.7, and *w* = 0.9. The buyer proposes the initial offer, and the seller continues to negotiate, with the discount rate 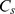 and 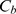 changing within the range of 0.5–1 and a minimum adjustment size of 0.025. The parameters in the control group without learning are the same as in the above-mentioned group except that agents in the control group do not adjust the probabilities of strategies.

The second experiment explores the impact of the weights of historical information on the negotiation result and strategy convergence. The adjustment range of *w* is 0.2–0.9, and the size is 0.1. Other parameters are the same as in the first experiment. We observe the negotiation result and calculate the related variables using the formulas in Table S1.

## Results

### Performance of the negotiation agents

**Figure 2:**
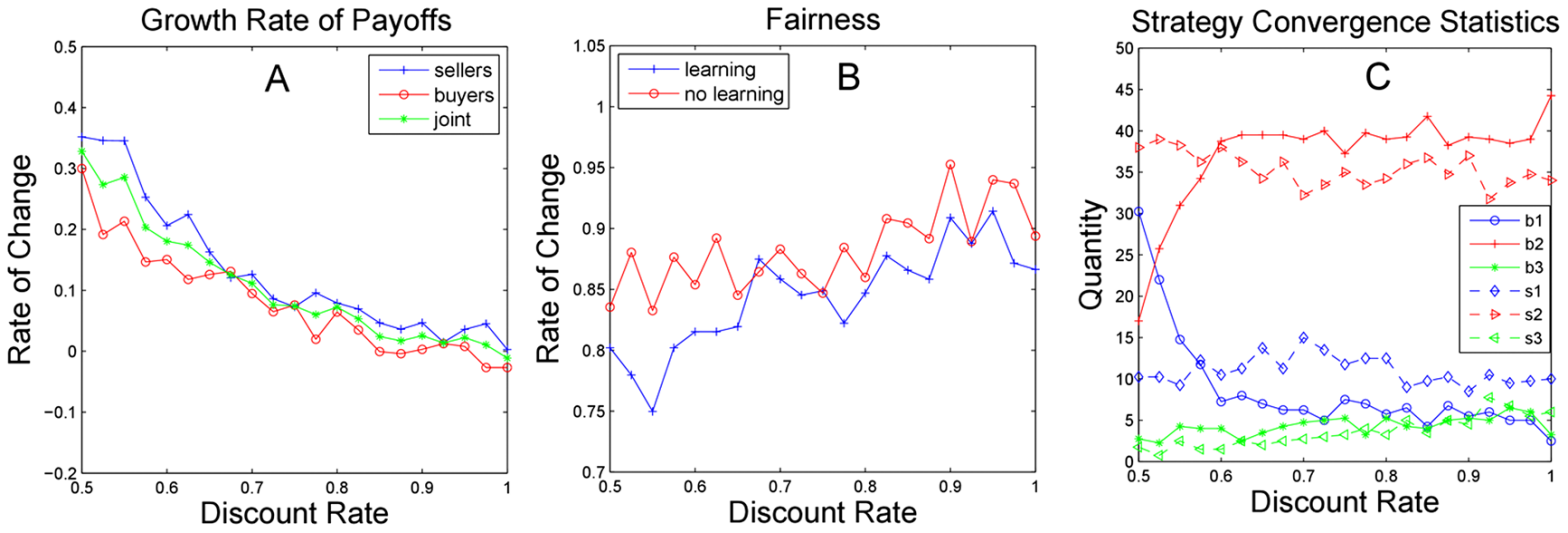
Negotiation performance of the agents. Figure 2A illustrates the growth rate of the average payoff with learning for buyers, sellers, and joint payoff compared to the growth rate without learning. Figure 2B illustrates the fairness comparison between the group with learning and the group without learning. Figure 2C shows the number of agents of each strategy in the convergence results, as every agent converges ultimately to a pure strategy.

The positive growth rate of the payoff in most cases means that the learning approach is beneficial to both buyers and sellers. As the discount rate decreases (see Figure 2A), the growth rate gradually increases. The reason may be that a lower discount rate encourages the intention of accepting an offer. The negotiation time is stable and only varies slightly with the change in discount rate, so it is not illustrated in Figure 2. Figure 2B illustrates the fairness with and without learning, where the noticeable difference between the two indicates that learning has changed the division of payoff between buyers and sellers. The distinction of the growth rate of payoffs in Figure 2A between the two populations is also the major reason for the variation of fairness with learning. Initialized with random subjective strategy probabilities, most agents finally converge to a cool-headed strategy. Only a small portion of agents converge to an anxious strategy or frugal strategy after periods of evolution. While the convergence results of the two populations are somewhat different, the patterns of strategy distribution are similar. The result shows that learning has changed the habit of strategy adoption, and most agents become sensible by using a cool-headed strategy despite lacking emotion.

### Impact of weight of historical information

**Figure 3:**
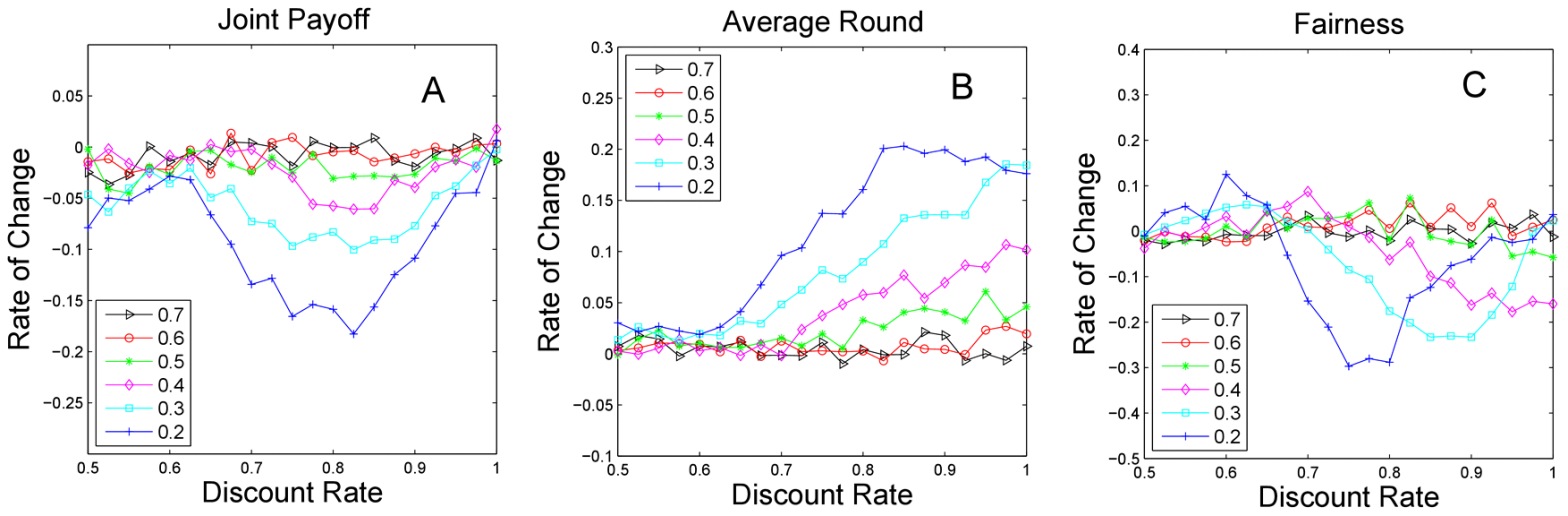
Impact of historical weight on payoff, time and fairness. Figures 3A, 3B and 3C illustrate the change in joint payoff, negotiation time, and fairness with different weights, compared with a weight of 0.8 as the baseline. Because the performance of agents with weights of 0.8 and 0.9 are very similar, the results for a weight of 0.9 are not displayed in Figure 3. To concisely demonstrate the result, we select the representative data with weights between 0.2 to 0.7 based on the benchmark of 0.8.

In Figure 3A, the joint payoff of buyers and sellers changes only slightly when the weight increases from 0.5 to 0.9. As the weight drops below 0.5, the payoff declines sharply with the decreasing discount rate and reaches a minimum at approximately 0.8. It is obvious that the agents who emphasize historical experience perform better than the agents who ignore it. In Figure 3B, the negotiation time remains stable when the weight is above 0.5 and rises obviously until the weight drops below 0.5. The discount rate in our model indicates that more rounds of negotiation lead to lower payoff and inefficiency of the market. In Figure 3C, there is only a minor fluctuation of fairness when the weight rises above 0.5, and fluctuation becomes noticeable when the weight drops below 0.5. It can be concluded that the profit division of the market becomes unfair if the agents rarely consider prior information.

To summarize, agents who value long-term experience achieve more stable and efficient performance such as joint payoff, negotiation time, and fairness.

**Figure 4:**
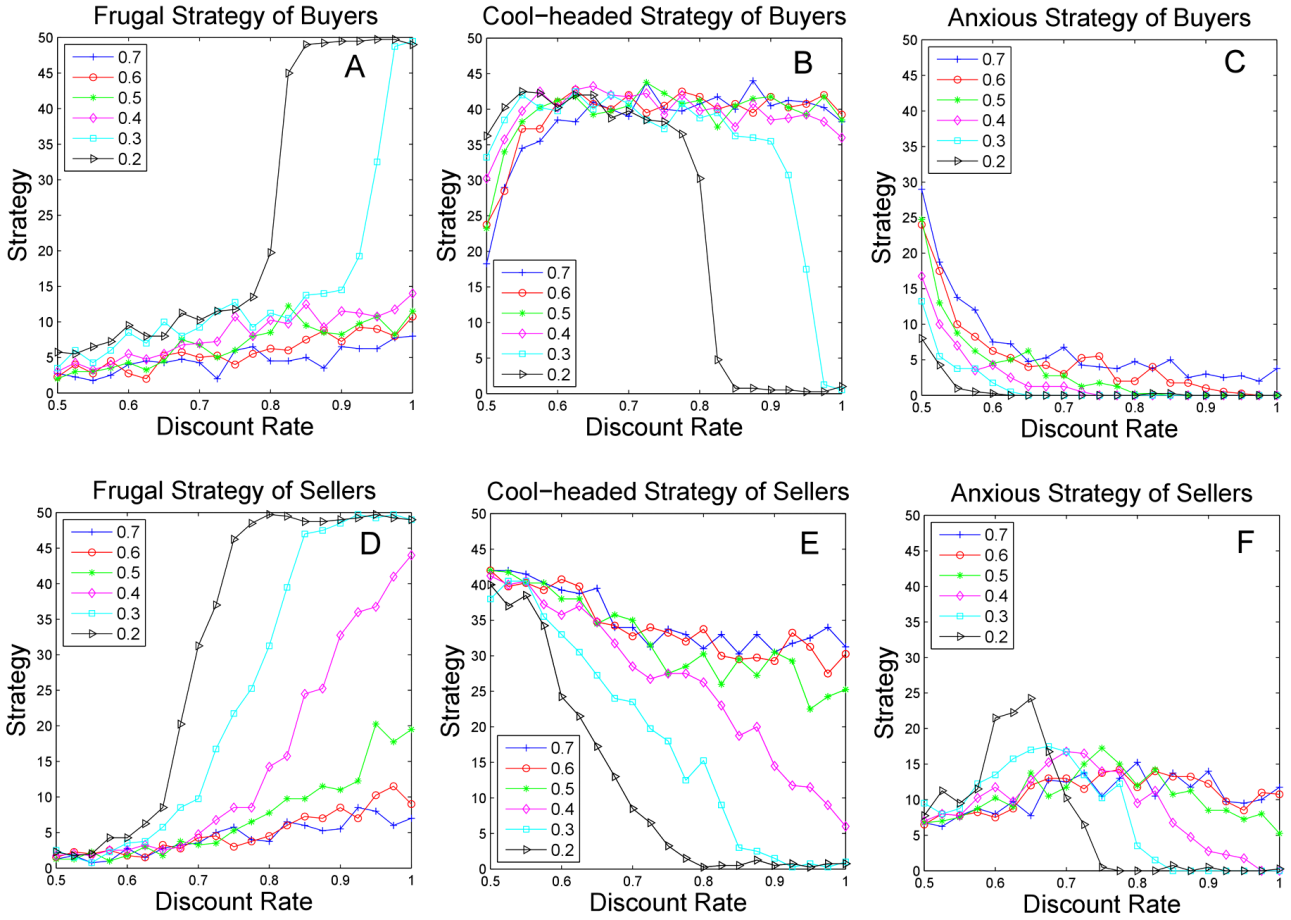
Impact of historical weight on the strategy convergence. Figures 4A, 4B and 4C illustrate the evolution results of the frugal, cool-headed, and anxious strategies of buyers using different weights. The Y axis represents the number of agents using each strategy in the population. Similarly, Figures 4D, 4E and 4F represent the corresponding results for sellers.

Figures 4A, 4B and 4C illustrate the impacts of the weight of historical information on buyers. The figures demonstrate that the convergence strategies remain stable when the weight is above 0.4 but vary significantly when the weight drops below 0.4. As the weight is high, the majority of the market adopts the cool-headed strategy, with the anxious and the frugal strategies as minorities. When the weight decreases, the advantage of the cool-headed strategy declines, while the frugal strategy increases and the anxious one remains stable. The results only change slightly with decreasing discount rate when the weight is high. In contrast, the results fluctuate substantially as the weight reaches a low level.

Figures 4D, 4E and 4F illustrate the impacts of the weight of historical information on the convergence of the frugal, cool-headed, and anxious strategies of the sellers, respectively. The general trend shows that the greediness of the agents rises notably when the weight decreases. Specifically, we find that the frugal strategy increases, while the cool-headed strategy simultaneously decreases. The frugal strategy is the minority when the weight is high and rises gradually when the weight drops, and it proves to be dominant at a high discount rate when the weight drops to approximately 0.2(see Figure 4D). The cool-headed strategy shows the opposite trend to the frugal one and declines as the weight decreases (see Figure 4E). As the discount rate decreases, the frugal strategy decreases, and the cool-headed strategy increases. The cool-headed strategy becomes dominant when the discount rate is very low, while the frugal strategy is a tiny minority. This result indicates that the low discount rate's leading to less greediness in agents may be caused by the time pressure to accept offers more actively. The anxious strategy remains at a low level when the weight is high and fluctuates substantially when the weight is below 0.5 (see Figure 4F). In conclusion, the strategy convergence remains stable and the cool-headed strategy has a noticeable advantage when the weight of historical information is high. The greediness of the whole market rises as the weight drops.

To summarize, the impacts of weight on convergence strategies are different between buyers and sellers. The strategies of sellers vary gradually with the weight, while the strategies of the buyers remain relatively stable when the weight is above 0.5 and change rapidly when the weight falls below 0.5. Because the only distinction between the two populations is the order of proposing the first offer, the asymmetry may arise from this factor. Therefore, the offering order not only affects the division of the payoff between buyers and sellers but also gives rise to the difference in the strategy distribution between them. The agents update their strategy probability after a period of negotiation during which the costs of agents remain stable, and therefore the frugal strategy achieves a higher payoff. The agents use a more greedy strategy when the weight is lower. Our results show the following: (1) myopic adjustment leads to a more frugal strategy with less concession, and (2) the algorithm with reinforcement learning results in a more cool-headed strategy with more efficient performance in the overall population, such as less negotiation time and less fluctuation in fairness.

## Discussion

The evolutionary approach is effective in investigating the collective behavior of the population in the long term. However, human learning involves more initiative than biological evolution, and therefore, we have integrated reinforcement learning with replicator dynamics to investigate negotiation behavior. Negotiation strategies are generally complex, and a new strategy type is created by design instead of mutation. In the genetic algorithm, the population of each generation is created by passive selection and reproduction [28]–[30], but agents representing humans usually will not depart the market even if they suffer from occasional loss in negotiation. Rather, they make decisions using initiative and accumulate experience through multiple periods of negotiation. To this end, this model evaluates the rewards of strategies by assigning weights to historical payoffs as well as to current ones. As a result, our learning pattern incorporating replicator dynamics differs from classic reinforcement learning, which determines the probability of strategies in proportion to the rewards [31]. Reinforcement learning has many models that differ from each other in details such as the probability determination rules. Rajiv Sarin and Farshid Vahid design a simple reinforcement learning model without probability, in which the agents choose the strategy with the highest reward instead of through subjective probability [32]. Borgers believes that individual learning is a process of idea evolution as well as habit formation and proves that a continuous-time reinforcement learning of an individual converges to equilibrium of replicator dynamics [19]. We adopt replicator dynamics as the strategy adjustment rule and incorporate reinforcement learning into the payoff evaluation. The simulation results suggest that agents using this new learning model achieve higher payoff, shorter negotiation time, and more stable fairness than agents using the classic evolutionary approach.

This paper presents two experiments designed to study the general performance of agents and the impacts of the weight of historical information on the negotiation result and convergence strategy. The results indicate that in most cases, learning increases the payoff of both buyers and sellers. Thus, the learning pattern is beneficial to both sides, and the growth rate rises with decreasing discount rate. Ravindra Krovi et al. [33] compare the payoff and fairness when one or two variables are controlled. However, they examine the evolution of offer instead of strategy, which is different from this paper. We evaluate the learning effect from the perspective of the population instead of the individual agent.

In addition to the market efficiency and fairness, we also consider long-term strategy evolution. In our model, all the agents converge to pure strategy and form stable habits. Although heterogeneous agents have different reservation values and initial states regarding strategies, the strategy distribution of the whole market is relatively stable, and the convergent results vary slightly with the initial settings. Noyda Matos et al. [10] allow the agents to use mixed strategies, but the proportion of each strategy is similar, and there is no dominant strategy. In our model, the majority is the cool-headed strategy, which means that the agents become rational by learning in the long run, although we do not consider psychological factors.

The classic evolutionary dynamics without considering historical information, including replicator dynamics and best response dynamics, are myopic adjustment dynamics [34]–[36]. The strategy adjustment in our model is not myopic because we have incorporated historical information into the evaluation. The simulation results indicate that the learning pattern focusing more heavily on historical information achieves better performance than its myopic counterparts. If agents ignore previous experience, their payoff declines, and simultaneously, negotiation time increases and fairness fluctuates substantially. The weight of prior experience also affects the strategy equilibrium, which includes mostly the cool-headed strategy and minor others.

This model still has a few limitations, such as the simple time-dependent concession function. We will consider more complex strategies such as behavior-dependent and resource-dependent concession functions in the future.

## Supporting Information

Table S1 **Variables and formulas.** Average payoff of buyer, average payoff of seller, joint payoff, average round and fairness are calculated with the corresponding formulas.
(DOCX)
