# Supplementary material for: Evolution with Reinforcement Learning in Negotiation
Source: PLoS One. 2014 Jul 21;9(7):e102840. doi: 10.1371/journal.pone.0102840 (PMC4105407; doi:10.1371/journal.pone.0102840)
Supplement: Table S1 — Variables and formulas. Average payoff of buyer, average payoff of seller, joint payoff, average round and fairness are calculated with the corresponding formulas. (DOCX) [file pone.0102840.s001.docx]

**Table S1.** Variables and formulas

| ***VARIALBLES*** | ***FORMULARS*** |
| --- | --- |
| Average payoff of buyer | $\sum_{i=1}^{N} (\frac{R_{b}^{i}-P^{i}}{N})$ |
| Average payoff of seller | $\sum_{i=1}^{N} (\frac{P^{i}-R_{s}^{i}}{N})$ |
| Joint payoff | $\sum_{i=1}^{N} (\frac{R_{b}^{i}-R_{s}^{i}}{N})$ |
| Average round | $\sum_{i=1}^{N} \frac{t^{i}}{N}$ |
| Fairness | $\frac{\sum_{i=1}^{N} (\frac{R_{b}^{i}-P^{i}}{P^{i}-R_{s}^{i}})}{N}$ |

Note: N is the negotiation amount, *i* the period, $t^{i}$the round of a successful negotiation and $P^{i}$ the transaction price.
